# Dietary saturated fatty acid palmitate promotes cartilage lesions and activates the unfolded protein response pathway in mouse knee joints

**DOI:** 10.1371/journal.pone.0247237

**Published:** 2021-02-22

**Authors:** Li Tan, Lindsey R. Harper, Alexandra Armstrong, Cathy S. Carlson, Raghunatha R. Yammani

**Affiliations:** 1 Department of Internal Medicine, Section of Molecular Medicine, Wake Forest School of Medicine, Winston-Salem, NC, United States of America; 2 Department of Veterinary Clinical Sciences, College of Veterinary Medicine, University of Minnesota, St. Paul, MN, United States of America; Wayne State University, UNITED STATES

## Abstract

Increased intake of dietary saturated fatty acids has been linked to obesity and the development of Osteoarthritis (OA). However, the mechanism by which these fats promote cartilage degradation and the development of OA is not clearly understood. Here, we report the effects of consumption of common dietary saturated and unsaturated fatty acids, palmitate and oleate, respectively, on body weight, metabolic factors, and knee articular cartilage in a mouse model of diet-induced obesity. Mice fed on a diet rich in saturated or unsaturated fatty acid gained an equal amount of weight; however, mice fed a palmitate diet, but not a control or oleate diet, exhibited more cartilage lesions and increased expression of 1) unfolded protein response (UPR)/endoplasmic reticulum (ER) stress markers including BIP, P-IRE1α, XBP1, ATF4, and CHOP; 2) apoptosis markers CC3 and C-PARP; and 3) negative cell survival regulators Nupr1 and TRB3, in knee articular cartilage. Palmitate-induced apoptosis was confirmed by TUNEL staining. Likewise, dietary palmitate was also increased the circulatory levels of classic proinflammatory cytokines, including IL-6 and TNF-α. Taken together, our results demonstrate that increased weight gain is not sufficient for the development of obesity-linked OA and suggest that dietary palmitate promotes UPR/ER stress and cartilage lesions in mouse knee joints. This study validates our previous in vitro findings and suggests that ER stress could be the critical metabolic factor contributing to the development of diet/obesity induced OA.

## Introduction

Osteoarthritis (OA) is the most common degenerative disease of joints, affecting nearly 27 million people in the United States; the prevalence of OA is expected to double by 2030 [1, 2]. OA is a disease of the entire joint, with the involvement of articular cartilage, subchondral bone, meniscus, and soft tissues (ligaments and tendons) during the progression of the disease [3]. Obesity is one of the most prevalent diseases globally [4] and is a major risk factor for developing OA, particularly in the knee joint [5]. Traditionally, the increased mechanical load on the joints alone, due to excess body weight, was accepted as the explanation for obesity-associated OA [6]. However, since obesity also increases the OA risk for non-weight bearing joints (e.g., hand) [5], increased weight alone is not sufficient to explain the relationship between OA and obesity.

Emerging evidence suggests that chronic inflammation associated with obesity plays a significant role in the pathogenesis of obesity-linked OA [7, 8]. Under obesity-associated mechanical stress, adipose and joint components such as chondrocytes and the infrapatellar fat pad are capable of producing multiple proinflammatory cytokines, including interleukin-1 (IL-1), IL-6, IL-10, IL-17, and tumor necrosis factor-α (TNF-α), to induce low-grade inflammation and promote cartilage matrix degradation leading to OA development [8, 9]. However, the relative contributions to OA of increased mechanical load and local and systemic inflammation occurring with obesity or its associated factors remain unclear.

Increased intake of a diet rich in fat is associated with increased plasma levels of circulating free fatty acids (FAs) [10]. Free FAs are divided into saturated and unsaturated FAs based on the presence or absence of a double bond in their molecular structure that confers their biological activity [11]. Saturated FAs are the dominant form of lipids in plasma and usually are found in mammalian animal fats, including lard. Besides providing energy, membrane fluidity, and lipid storage in tissues, saturated FAs also activate proinflammatory pathways and play a significant role in several human pathologies, including type 2 diabetes, metabolic syndrome, and obesity [12, 13]. Unsaturated FAs can be further classified into monounsaturated and polyunsaturated FAs depending on the number of double bonds that they possess [11]. Interestingly, substituting saturated FAs with monounsaturated FAs in controlled and isoenergetic diets significantly enhanced insulin sensitivity in healthy humans [14], suggesting that a change in dietary fat quality alone could have significant impacts on obesity-linked diseases.

Increased levels of free FAs are associated with increased severity of cartilage lesions in OA [15]. Accumulation of palmitate and oleate, the most abundant saturated and monounsaturated FAs in human tissues, respectively, have been found in OA cartilage [15]. Interestingly, palmitate, but not oleate, induces chronic inflammation, and cell death in vitro [16]. Recently, we also demonstrated that palmitate, rather than oleate, induces endoplasmic reticulum (ER) stress and promotes apoptosis in both cultured chondrocytes and meniscus cells [17, 18]. These studies suggest that palmitate might be a key component in dietary fat that triggers inflammation and ER stress for obesity-linked OA.

It has been reported that ER stress is a link between obesity and the development of type 2 diabetes [19]. Obesity-induced ER stress also causes chronic inflammation in murine adipose tissue [20]. Moreover, ER stress appears to inhibit the function of insulin-like growth factor-1 for cartilage matrix biosynthesis in obese mice [21]. However, the role of ER stress in OA pathogenesis is not clearly established.

ER stress triggers unfolded protein response (UPR) pathways mediated by three axes of stress sensors: activating transcription factor 6 (ATF6), inositol-requiring enzyme 1 (IRE1), and protein kinase R-like ER kinase (PERK) [22, 23]. During unstressed conditions, these proteins are inactive and are bound to the ER chaperone, binding immunoglobulin protein (BIP, also called GRP78). Under stress conditions (e.g., accumulation of unfolded proteins), BIP releases itself from the sensors to bind unfolded proteins, thus activating the individual UPR pathways. When ER stress is severe and irrecoverable, all three axes of UPR pathways could induce C/EBP homologous protein (CHOP), a proapoptotic molecule, to eventually activate caspase-mediated apoptosis [24].

In the present study, we investigated the mechanism by which dietary palmitate promotes the development of OA. Mice fed with a novel iso-caloric diet that is supplemented with palmitate or oleate gained similar body weights during the length of the diet regimen and allowed us to precisely evaluate the role of a metabolic factor/component (e.g., palmitate) in cartilage degradation and development of OA while excluding the influence of increased mechanical burden on joints due to excess gain in body weight. We found that dietary palmitate, but not oleate, induced ER stress and chondrocyte apoptosis and promoted cartilage degradation and OA. To the best of our knowledge, this is the first report to demonstrate the mechanistic role of palmitate in the initiation of OA *in vivo*.

## Materials and methods

### Animal studies

Ten-week-old male C57Bl/6J mice (n = 45) were housed in the Wake Forest School of Medicine Vivarium under a 12-h dark/light cycle and were provided standard chow diet and water ad libitum. At 12 weeks of age, mice were divided into 3 groups (n = 15 per diet group) and were provided a special diet for 20 weeks. Group 1 mice were maintained on a control diet (corn oil; 20% calories from oil/fat). Mice in Groups 2 and 3 were maintained either a high-palmitate (20% calories from oil/fat) diet (palmitate) or a diet containing 20% calories from oleate (oleate). The special diets are made in the diet kitchen in the Department of Pathology at Wake Forest School of Medicine, as described previously [25]. The quality and quantity of lipids in the diet were analyzed before the start of treatment and at the end of treatment by the Wake Forest University Botanical Center Lipid core lab as previously described [26, 27]. All experiments were approved by the Wake Forest School of Medicine Animal Care and Use Committee. Each mouse was weighed at weeks 0, 10, and 20, respectively. At the end of the study, mice were sacrificed by CO_2_ asphyxiation followed by cervical dislocation, and knee joints were routinely fixed in 10% formalin, decalcified in 10% EDTA, processed, and embedded in paraffin, and serially sectioned in a midcoronal plane at a thickness of 4 μm. Sections from each joint were blinded for histological and immunohistochemical analyses.

### Histology

A single mid-coronal section from one knee per mouse was stained with hematoxylin and eosin (H&E) for histological assessment. Mouse joint measurements were made using the OsteoMeasure histomorphometry system (OsteoMetrics) as previously described [28], including articular cartilage area and thickness, number of viable chondrocytes, and articular cartilage structure score (ACS, grade 0–12). The ACS system scored the integrity of the articular cartilage on a scale of 0–12, where 0 represents normal healthy cartilage, and 12 represents full-thickness loss of articular cartilage across more than two-thirds of the surface scored. Synovial hyperplasia was also scored on a scale of 0–3, as described previously [29]. Briefly, a grade of 0 = 1–3 cell layers of synoviocytes, 1 = 4–6 cell layers of synoviocytes, 2 = 7–9 cell layers of synoviocytes, and 3 = 10 or more cell layers of synoviocytes.

### Immunohistochemistry

Paraffin-embedded sections were deparaffinized in a xylene substitute, Clear Advantage (Polysciences), rehydrated through a series of decreasing concentrations of ethanol, and washed with Tris-buffered saline (TBS). Antigen retrieval was achieved with proteinase K treatment for 5 min. Sections were washed with TBS, treated with 3% hydrogen peroxide for 15 min, washed with TBS, blocked with Vectastain^®^ normal goat serum for 15 min at room temperature, and incubated with primary antibody [1:50 dilution for CHOP; 1:100 dilutions for phosphorylated IRE1 alpha (P-IRE1α), activating transcription factor 4 (ATF4), and cleaved poly(ADP-ribose) polymerase (C-PARP); 1:200 dilutions for spliced X-box binding protein-1 (XBP1), nuclear protein 1 (Nupr1) and *tribbles* related protein-3 (TRB3); 1:500 dilution for BIP and cleaved caspase 3 (CC3)] in blocking serum overnight at 4°C. After washing with TBS, sections were incubated with biotinylated anti-rabbit secondary antibody for 30 min at room temperature, washed with TBS, and then incubated with Vectastain^®^ Elite ABC reagent for 30 min at room temperature. Sections were again washed with TBS, incubated with ImmPACT^TM^ NovaRED^TM^ peroxidase substrate (Vector Laboratories) for 5–30 min, washed with TBS, dehydrated and mounted. The following antibodies were used: rabbit polyclonal anti-BIP (ab21685), rabbit polyclonal anti-ATF4 (ab105383), rabbit monoclonal anti-CHOP (ab179823), and rabbit monoclonal anti-C-PARP (ab32064), all from Abcam; rabbit polyclonal anti-P-IRE1α (PA1-16927 from Thermo Fisher Scientific), rabbit polyclonal anti-XBP1 (AP07389PU-N from OriGene Technologies); rabbit monoclonal anti-CC3 (9664 from Cell Signaling Technology), rabbit polyclonal anti-Nupr1 (bs-7106R from Bioss), and rabbit polyclonal anti-TRB3 (13300-1-AP from Proteintech). At least three immunohistochemical images/regions containing 40–60 chondrocytes in the mouse knee cartilage were quantified using Adobe Photoshop CS6 (version 13.0) with correction for cell numbers. Briefly, an image was first converted to a black & white image and then inverted into a white & black image. The threshold value for cell staining intensity was determined in the images of a control group to avoid light from the background, and the same value was then applied to images in the palmitate and oleate groups. Multiple regions containing 40–60 chondrocytes were selected and their total pixel numbers were measured and then divided by the cell numbers in the same region. As a negative control, immunohistochemical staining with only secondary antibody by replacing a primary antibody with the blocking serum essentially showed no staining for any of the groups.

### TUNEL staining

Chondrocyte apoptosis was further confirmed by TUNEL assay [30]. Paraffin-embedded sections were TUNEL stained according to manufacturer (Abcam) protocol and then visualized using an Echo revolve fluorescence microscope. We used a single section from the right knee/mouse (n = 3) to perform the TUNEL assay. The TUNEL staining data were also quantified, as described above.

### Cirasoft analysis

Mice were fed a control, palmitate, or oleate diet for 20 weeks. Blood was collected from each mouse and centrifuged to obtain serum samples. The serum samples within each diet group were then pooled (5 mice/pool) due to limited volume. The pooled samples were assayed to evaluate the expression of cytokines, including IL-6, IL-10, TNF-α, IFN-γ, IL-1β, IL-12p70, and IL-17, according to Multiplex Planar Array Kit Cytokine Panel 1. Using a 96-well plate spotted with target analysts of interest, pooled serum samples were diluted with assay diluent to achieve 1:1 and 1:10 dilutions and incubated with Biotinylated labeled antibodies for immunocomplex detection. A chemiluminescent substrate was then added just prior to ultra-sensitive camera imaging to measure the signal intensity produced by each analyst. The obtained data were analyzed by Quanterix® SP-X^TM^ Imaging and Analysis System using Aushon Cirasoft Analyst Software.

### Statistical analysis

Data are expressed as mean ± standard deviation. Statistical analysis of data was performed using GraphPad Prism 8 (version 8.2.0) as follows: non-parametric Mann-Whitney test with exact P values for ACS and synovial hyperplasia scores, and two-tailed unpaired *t*-test with exact P values for chondrocyte counts per cartilage area, proinflammatory cytokine levels, and quantifications of immunohistochemical and TUNEL data. The results were considered statistically significant at a value of P ≤ 0.05. For multiple comparisons, the significance level was adjusted by Bonferroni correction using the following formula: P ≤ 0.05/number of comparisons.

## Results

### Dietary palmitate induces cartilage lesions and synovial hyperplasia in a mouse OA model

**Fig 1:**
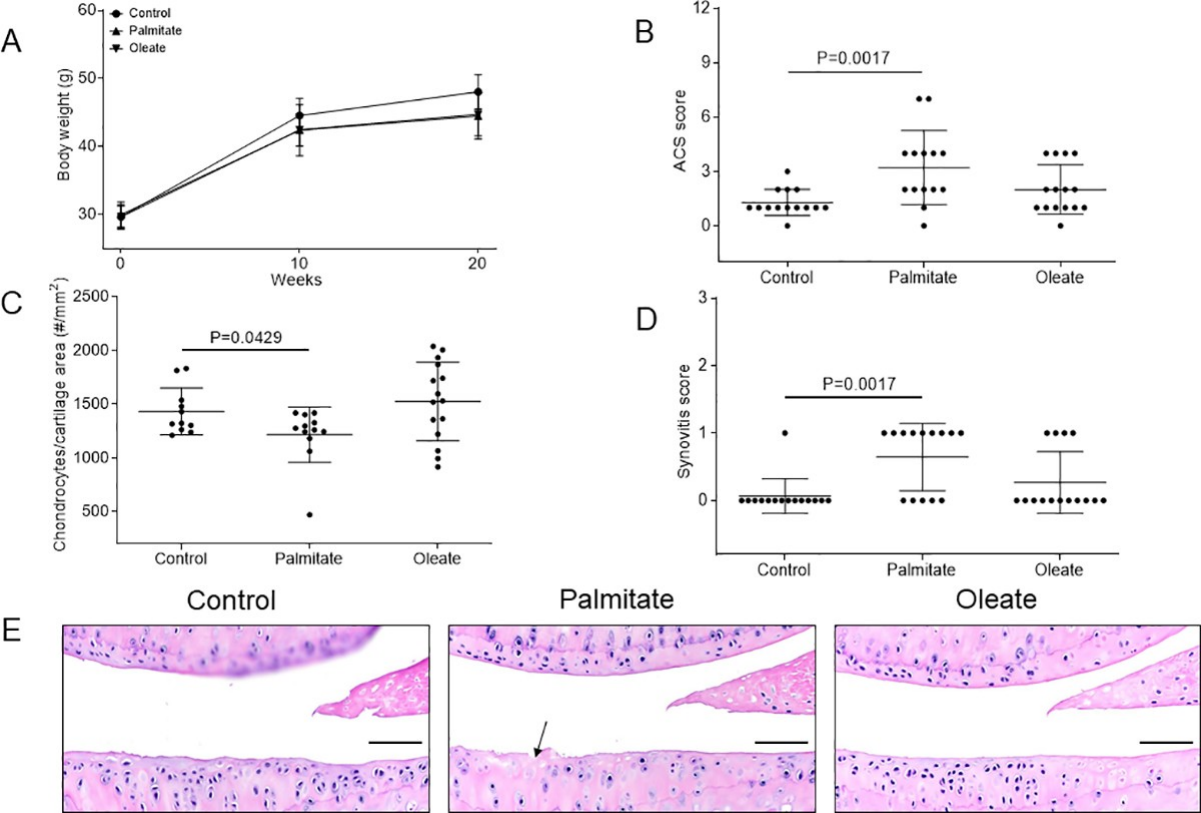
Dietary palmitate induces cartilage lesion and synovial hyperplasia in a mouse OA model. Mice were fed with a control, palmitate, or oleate diet (n = 15 per diet group) for 20 weeks. (A) Body weights of mice were measured at weeks 0, 10, and 20, respectively. Values are mean ± standard deviation. Mouse knee joints were then collected, processed and sectioned (one section from one knee per mouse) for histological analyses including (B) ACS score on medial tibial plateau, (C) viable chondrocyte counts per cartilage area, and (D) synovial hyperplasia score, with (E) hematoxylin and eosin (H&E) staining of mouse knee joint sections showing viable chondrocytes with blue-stained nuclei. Black arrow represents fibrillation of the articular cartilage. Scale bar, 50 μm.

Increased body weight due to obesity is thought to play a major role in the development of obesity-linked OA. To address this issue, we fed male C57Bl/6J mice with a control diet and diet rich in palmitate or oleate diet for 20 weeks and measured the mouse body weights in each diet group throughout the study. With our special iso-caloric diets, mice in all diet groups exhibited a similar weight gain (about 50% total increase) throughout the diet regimen. The palmitate and oleate diet groups maintained nearly identical average body weights during the entire experiment (Fig 1A).

High- fat diet has been shown to induce cartilage lesions in mouse OA models [31–33]. To determine if the composition of dietary fatty acids contributed to OA development *in vivo*, the knee joints from mice on the control, palmitate, and oleate diets were analyzed for OA severity. Histological evaluation of hematoxylin and eosin (H&E) sections revealed statistically significant increases in articular cartilage structure (ACS) scores in the joints of the mice on the palmitate diet (mean severity of 3.2±2.0 out of 12, n = 14), but not on the oleate diet (mean severity of 2.0±1.3 out of 12, n = 15), compared to mice on the control diet (mean severity of 1.3±0.7 out of 12, n = 14; Fig 1B and 1E) [28], suggesting that dietary palmitate, but not oleate, induces articular cartilage lesion in mouse knee joints without excess increases in body weight as compared to controls. Moreover, mice on the palmitate diet showed lower viable chondrocyte counts per cartilage area compared to mice on the control diet (Fig 1C). Furthermore, mice on a palmitate diet also showed higher synovial hyperplasia scores compared to mice on the control diet (Fig 1D), suggestive of palmitate-induced synovial reaction. These results collectively suggest that dietary palmitate promotes chondrocyte cell death, mild OA, and synovial hyperplasia in this mouse model.

### Dietary palmitate induces UPR signaling / ER stress in mouse knee cartilage

**Fig 2:**
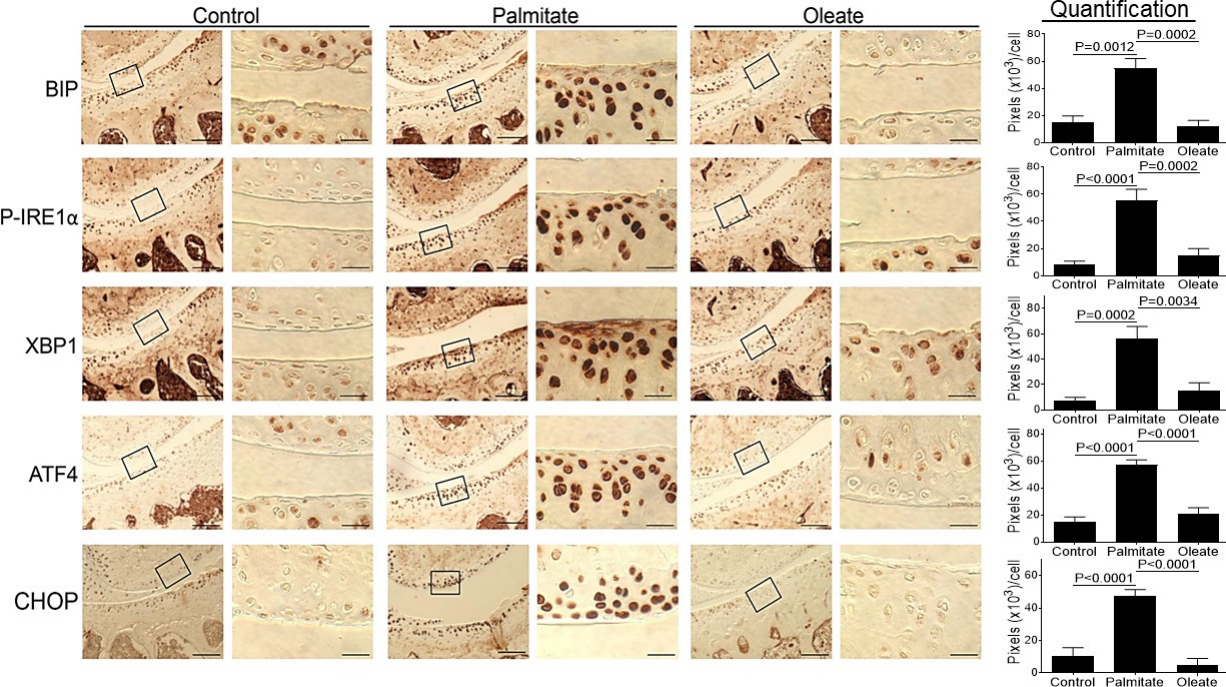
Dietary palmitate induces ER stress in mouse knee cartilage. Mice were fed with a control, palmitate, or oleate diet (n = 15 per diet group) for 20 weeks. Joints were collected, processed and sectioned (one section knee/mouse) for immunohistochemical staining. Coronal sections of mouse knee joints were analyzed for ER stress markers including BIP, P-IRE1α, XBP1, ATF4, and CHOP. Images on left panels in each pair of columns are of low magnification (Scale bars: 100 μm), and the tibia is in the lower half of images. The areas inside the small rectangles were magnified and displayed in right panels (scale bars: 20 μm). All immunohistochemical data were quantified with correction for cell numbers and statistically analyzed. The bars were obtained from the analysis of 3 mice per group (n = 3), and the data are expressed as the average pixel numbers (×10^3^) per cell ± standard deviation.

Our *in vitro* studies have shown that treatment of chondrocytes or meniscus cells with palmitate induced ER stress and activated UPR signaling *in vitro* [17, 18, 21]. To test if dietary palmitate induces ER stress in mouse knee cartilage, paraffin-embedded knee joint sections of mice fed a palmitate diet were prepared and evaluated immunohistochemically for multiple ER stress protein markers. Our data showed statistically significant increased expression of ER stress markers and UPR signaling molecules, including BIP, phosphorylated IRE1 alpha (P-IRE1α), spliced X-box binding protein-1 (XBP1), activating transcription factor 4 (ATF4) and CHOP, in the joints of the mice fed a palmitate diet compared to mice fed a control or oleate diet (Fig 2), demonstrating that dietary palmitate rather than oleate induces ER stress in mouse knee joints, confirming our previous observations in cell culture models.

### Dietary palmitate induces chondrocyte apoptosis in mouse knee cartilage

**Fig 3:**
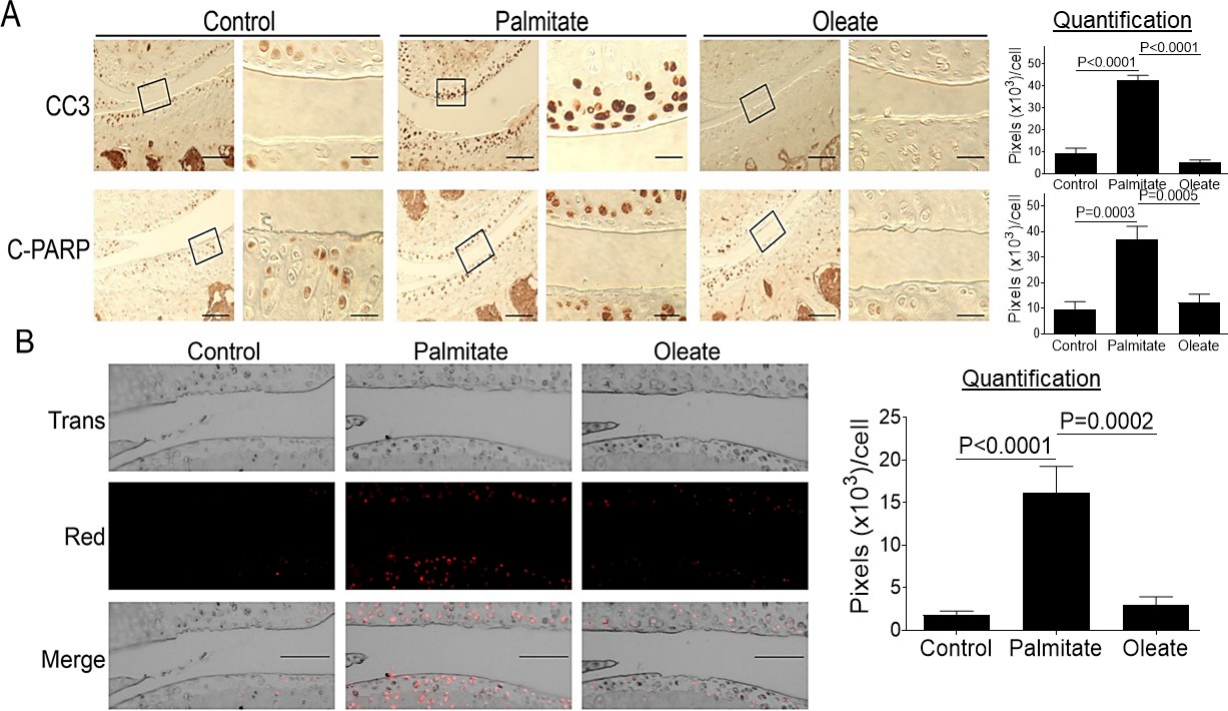
Dietary palmitate induces chondrocyte apoptosis in mouse knee cartilage. Mice were fed a control, palmitate, or oleate diet (n = 15 per diet group) for 20 weeks. Joints were collected, processed, and sectioned. We used a single section from the right knee/mouse (n = 3) for immunohistochemical and TUNEL staining. (A) Coronal sections of mouse knee joints were analyzed immunohistochemically for apoptosis markers CC3 and C-PARP. Images on left panels in each pair of columns are of low magnification (Scale bars: 100 μm), and the tibia is in the lower half of images. The areas inside the small rectangles were magnified and displayed in right panels (scale bars: 20 μm). (B) Coronal sections of mouse knee joints were evaluated by TUNEL staining. Scale bar, 100 μm. All immunohistochemical and TUNEL data were quantified with correction for cell numbers and statistically analyzed. The bars were obtained from the analysis of 3 mice per group (n = 3), and the data are expressed as the average pixel numbers (×10^3^) per cell ± standard deviation.

Chronic and sustained ER stress has been reported to induce caspase-mediated apoptosis [22, 34]. As was recently demonstrated by our work and that of other researchers, palmitate induces caspase-mediated apoptosis in cultured chondrocytes or meniscus cells [16–18], leading us to investigate whether palmitate also activates caspase-mediated apoptosis *in vivo*. Immunohistochemical analyses of knee joint sections showed statistically significant increased expression of two apoptosis markers, cleaved caspase 3 (CC3) and cleaved poly(ADP-ribose) polymerase (C-PARP), in knee cartilage from mice fed a palmitate diet compared to mice fed a control or oleate diet (Fig 3A). The palmitate-induced apoptosis was confirmed by TUNEL staining as a functional assay to assess apoptosis-linked DNA fragmentation [30] (Fig 3B), consistent with previous *in vitro* findings [17, 18].

### Dietary palmitate induces increased expression of Nupr1 and TRB3 in mouse knee cartilage

**Fig 4:**
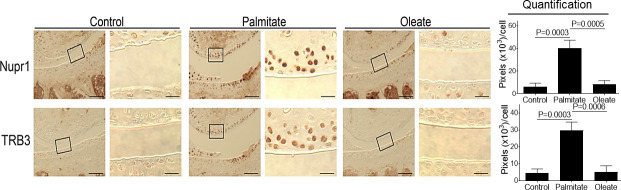
Dietary palmitate induces increased expression of Nupr1 and TRB3 in mouse knee cartilage. Mice were fed a control, palmitate, or oleate diet (n = 15 per diet group) for 20 weeks. Joints were collected, processed and sectioned (one section knee/mouse) for immunohistochemical staining. Coronal sections of mouse knee joints were analyzed for Nupr1 and TRB3. Images on left panels in each pair of columns are of low magnification (Scale bars: 100 μm), and the tibia is in the lower half of images. The areas inside the small rectangles were magnified and displayed in right panels (scale bars: 20 μm). All immunohistochemical data were quantified with correction for cell numbers and statistically analyzed. The bars were obtained from the analysis of 3 mice per group (n = 3), and the data are expressed as the average pixel numbers (×10^3^) per cell ± standard deviation.

Palmitate also induces the expression of nuclear protein 1 (Nupr1) and *tribbles* related protein-3 (TRB3) *in vitro* that are known to play a critical role in cell survival and apoptosis [17, 35, 36]. Since elevated expression of both proteins has been found in human osteoarthritic cartilage [37, 38], we determined whether dietary palmitate induces increased expression of Nupr1 and TRB3 in mouse knee cartilage. Compared to mice on control or oleate diets, mice in the palmitate diet group showed increased expression of both Nupr1 and TRB3 in knee cartilage (Fig 4), demonstrating that dietary palmitate induces protein expression of Nupr1 and TRB3 in mouse knee joints, confirming observations from our recent *in vitro* study [17].

### Dietary palmitate induces proinflammatory cytokines in mice

**Fig 5:**
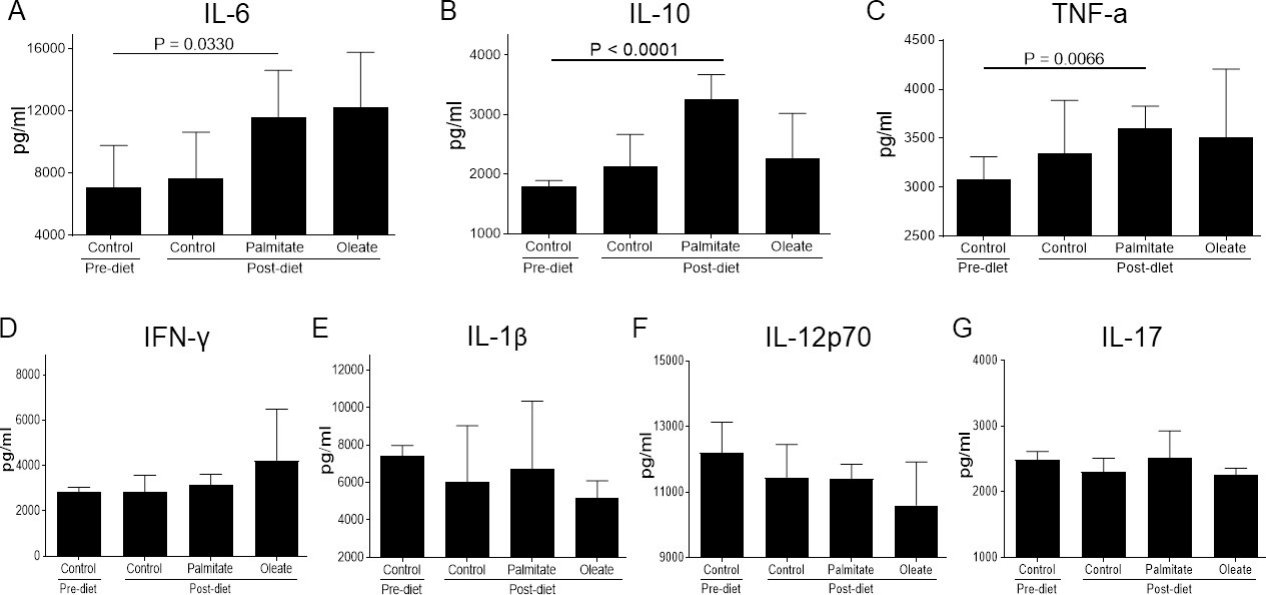
Dietary palmitate induces proinflammatory cytokines in mice. Mice were fed a control, palmitate, or oleate diet (n = 15 per diet group) for 20 weeks. Blood was collected from each mouse and pooled (5 mice/pool) due to limited volume. Cirasoft analysis of the pooled serum samples was then performed to measure the level of cytokines including (A) IL-6, (B) IL-10, (C) TNF-α, (D) IFN-γ, (E) IL-1β, (F) IL-12p70, and (G) IL-17. Data are expressed in pg/ml.

Since palmitate also induces proinflammatory cytokine expression in cultured human articular chondrocytes and cartilage explants *in vitro* [16], we examined whether dietary palmitate induces proinflammatory cytokines *in vivo*. Cirasoft analysis of serum from mice fed a palmitate diet showed elevated expression of cytokines IL-6, IL-10, and TNF-α compared to their pre-diet levels (Fig 5A–5C). However, there was no difference in the expression of IFN-γ, IL-1β, IL-12p70, and IL-17 (Fig 5D–5G).

## Discussion

**Fig 6:**
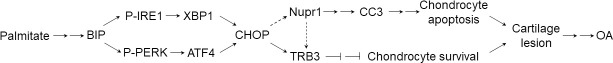
Model of palmitate-induced ER stress and chondrocyte apoptosis in mouse knee joints. Dietary palmitate induces ER stress in mouse knee joints. Under ER stress, BIP is released from IRE1 to trigger activation of IRE1 via autophosphorylation (P-IRE1) which further activates the expression of XBP1. Similarly, PERK is autophosphorylated (P-PERK) following the release of BIP thus inducing the ATF4 expression. Both XBP1 and ATF4 induce CHOP expression which further induces the expression of Nupr1 to elicit CC3-mediated chondrocyte apoptosis. Both CHOP and Nupr1 could induce TRB3 expression to inhibit chondrocyte survival. →, single-step stimulation;, putative single-step stimulation; → →, multistep stimulations; ⊥ ⊥, multistep inhibitions.

Traditionally, development of obesity-linked OA is attributed to bio-mechanical factors (biomechanical stress on joints due to increased body weight) [6]. However, increasing evidence suggests multiple metabolic factors associated with high-fat diet/obesity play an important role in the development of OA that go beyond the direct biomechanical stress due to increased body weight [32, 33, 39]. However, delineating these two factors has been a challenge. In this study, we found that mice fed a novel iso-caloric diet supplemented with palmitate or oleate gained similar body weight; however, mice on dietary palmitate developed more cartilage lesions in the knee joints compared to control (placebo) or oleate diet. Interestingly, palmitate diet increased the expression of ER stress and apoptotic markers in the articular cartilage of mouse knee joints. Furthermore, the palmitate diet also increased the serum proinflammatory markers, including IL-6 and TNFα. TUNEL staining of mouse knee joints confirmed chondrocyte death. Chondrocyte death has been associated with the severity of cartilage lesions and the development of OA [40]. Taken together, our data suggest UPR signaling/ER stress could be a key metabolic factor that could play a role in obesity-linked OA (Fig 6). Our findings also suggest that the biomechanical factors due to increased body weight were not sufficient to induce cartilage lesions and the development of obesity-linked OA. However, a cumulative effect of inflammation and increased mechanical factors could still play a role in obesity-linked OA.

We and others have shown that palmitate induces apoptosis and proinflammatory markers, including IL-6 in chondrocytes and meniscus *in vitro* studies [16–18, 41]. We also reported that palmitate inhibits proteoglycan synthesis in chondrocytes [21]. Rats fed a high–fat diet rich in saturated FAs have been shown to develop OA-like cartilage lesions [42], and mice on a diet rich in omega-6 FA also showed increased OA-like cartilage lesions [43]; however, the molecular mechanism involved was not determined.

Here, we identified ER stress and UPR pathway as a potential mechanism for palmitate mediated chondrocyte apoptosis based on our findings and those of others [17, 22–24]. Feeding mice a diet supplemented with palmitate causes lipid toxicity in joint tissues and triggers ER stress. Under severe ER stress, BIP is released from IRE1 and PERK to activate the individual UPR signaling axes. The release of BIP from IRE1 results in homodimerization and activation of IRE1 via autophosphorylation (P-IRE1), which further activates the expression of spliced XBP1. Likewise, disassociation of BIP from PERK leads to activation of PERK by autophosphorylation (P-PERK), thereby inducing expression of the protein ATF4. Under chronic and severe ER stress, both XBP1 and ATF4 could induce the proapoptotic molecule CHOP [23, 44]. Elevated CHOP expression further induces the expression of Nupr1 to activate CC3-mediated chondrocyte apoptosis [17]. Both CHOP and Nupr1 could also induce the expression of TRB3 to further inhibit chondrocyte survival [17, 45–47]. Taken together, dietary palmitate may play a role in inducing ER stress, chondrocyte apoptosis, and development of cartilage lesions in mouse knee joints. It is worth mentioning that the third axis of UPR signaling mediated by ATF6 is not shown in this model merely because we could not differentiate the full-length inactive ATF6 from its active form of truncated ATF6 using an ATF6 antibody for immunohistochemistry [22].

Our results demonstrate that dietary palmitate appears to induce two crucial metabolic factors, inflammation and UPR signaling/ER stress, both contributing to obesity-associated OA. The nature of the relationship between inflammation and ER stress in the development of OA needs further research to be fully understood. Palmitate has been recently found to induce inflammation through toll-like receptor 4-dependent priming and altered cellular metabolism, eventually activating c-Jun N-terminal kinase (JNK) [48], which is actually one of the important downstream targets of P-IRE1 in ER stress/UPR pathways [23, 34]. Furthermore, ER-stress-induced TRB3 is capable of promoting β-cell apoptosis via the NF-κB pathway [47], which plays an essential role in the regulation of obesity-linked inflammation. Therefore, both inflammation and ER stress may contribute at least in part through shared pathways to the development of obesity-linked OA.

Dietary palmitate also induced increased circulatory levels of IL6, TNF-α, and IL-10 in mice compared to pre-diet levels. However, no significant differences were observed between dietary groups after 20 weeks, although a clear uptrend (higher mean values) was observed for these cytokines (IL-6, TNF-α, and IL-10) with dietary palmitate compared to dietary oleate or control diet. It is possible that extending the diet regimen and/or increased sample size could have made our data more statistically significant. Interestingly, dietary palmitate increased circulatory IL-10 levels. IL-10 is usually considered to be an inhibitory cytokine that antagonizes the effects of proinflammatory cytokines such as IL-1, IL-6, and TNF-α [9, 49]. However, since IL-10 expression has also been found to be elevated in human osteoarthritic cartilage and both IL-10 and the IL-10 receptor are highly expressed in human fetal cartilage [50], it is possible that IL-10 might play another role in the regulation of chondrocyte metabolism beyond its immunological activity.

In conclusion, we have shown that dietary palmitate-induced UPR signaling/ ER stress, chondrocyte apoptosis, and cartilage lesions in a mouse model, validating our previous in vitro findings [17, 18]. We acknowledge that we have not provided evidence showing that pharmacological mitigation of ER stress reduces the palmitate-induced cartilage lesions and the development of OA, which is a limitation of this study. However, we recently showed that the administration of the general ER stress inhibitor 4-phenyl butyric acid (PBA) reduced high-fat diet-induced ER stress and chondrocyte apoptosis in mouse knee joints [51], supporting our hypothesis that palmitate-induced ER stress might be a key metabolic factor that promotes cartilage damage and could be targeted for obesity/diet-linked OA therapy.

## Supporting information

S1 File (DOCX)
