# Supplementary material for: Dietary saturated fatty acid palmitate promotes cartilage lesions and activates the unfolded protein response pathway in mouse knee joints
Source: PLoS One. 2021 Feb 22;16(2):e0247237. doi: 10.1371/journal.pone.0247237 (PMC7899342; doi:10.1371/journal.pone.0247237)
Supplement: S1 File — (DOCX) [file pone.0247237.s001.docx]

**S1 File.**

Dietary saturated fatty acid palmitate promotes cartilage lesions and activates the unfolded protein response pathway in mouse knee joints

Li Tan^1^, Lindsey R Harper ^2^, Alexandra Armstrong^2^, Cathy S. Carlson^2^, and Raghunatha R. Yammani^1*^

^1^ Section of Molecular Medicine, Department of Internal Medicine, Wake Forest School of Medicine, Winston-Salem, NC 27157, USA

^2^Department of Veterinary Population Medicine, College of Veterinary Medicine, University of Minnesota, St. Paul, MN 55108, USA

* Corresponding author

E-mail: [ryammnai@wakehealth.edu](mailto:ryammnai@wakehealth.edu) (RRY)

**S1 Table. Raw data used for Fig 1A.**

| **Week** | **Body weights (g) of mice in control group** | | | | | | | | | | | | | | |
| --- | --- | --- | --- | --- | --- | --- | --- | --- | --- | --- | --- | --- | --- | --- | --- |
| **0** | 30.6 | 28.3 | 29.3 | 29.6 | 29.2 | 26.7 | 32.4 | 32.5 | 30.3 | 29 | 28.1 | 28.5 | 30.9 | 30.1 |  |
| **10** | 47 | 42 | 42.9 | 47 | 44 | 40.9 | 46.8 | 48.6 | 47 | 44 | 43.1 | 41.2 | 46.5 | 42.8 |  |
| **20** | 51.6 | 45.6 | 46.4 | 49.5 | 46.5 | 44 | 50 | 52.8 | 49.3 | 47.5 | 45.3 | 49 | 49 | 46.2 |  |
| **Week** | **Body weights (g) of mice in palmitate group** | | | | | | | | | | | | | | |
| **0** | 30.1 | 27.7 | 30.8 | 28.1 | 31.1 | 30.4 | 32.2 | 28.6 | 28.3 | 29.9 | 34.4 | 30.3 | 28.6 | 27.9 | 31.5 |
| **10** | 44.5 | 39 | 43 | 38 | 46.5 | 40.1 | 33 | 42.5 | 43.5 | 45 | 46.4 | 45.1 | 40 | 43.5 | 46 |
| **20** | 48.6 | 42.7 | 42.6 | 40.4 | 48.6 | 44.2 | 40.5 | 45.9 | 43.7 | 44.8 | 50.4 | 43.5 | 41.5 | 40.8 | 49.4 |
| **Week** | **Body weights (g) of mice in oleate group** | | | | | | | | | | | | | | |
| **0** | 30.2 | 27 | 31.3 | 30.6 | 29 | 27 | 31.9 | 32.5 | 30.8 | 27.9 | 27.8 | 28.8 | 29.4 | 28.8 | 32 |
| **10** | 43 | 41 | 46.6 | 44 | 41.9 | 40 | 38 | 45 | 45.9 | 41 | 41 | 39.8 | 43 | 43.3 | 43.5 |
| **20** | 43.7 | 44.2 | 49.3 | 46.5 | 42.8 | 41.7 | 47.5 | 47.2 | 47.4 | 43 | 43.5 | 47 | 46.6 | 44.4 | 36.4 |

**S2 Table. Raw data used for Fig 1B.**

| **Group** | **Articular cartilage structure (ACS) score** | | | | | | | | | | | | | | |
| --- | --- | --- | --- | --- | --- | --- | --- | --- | --- | --- | --- | --- | --- | --- | --- |
| **Control** | 2 | 1 | 1 | 2 | 1 | 1 | 1 | 3 | 1 | 1 | 1 | 0 | 1 | 2 |  |
| **Palmitate** | 4 | 4 | 7 | 2 | 4 | 7 | 2 | 2 | 0 | 2 | 4 | 4 | 1 | 2 |  |
| **Oleate** | 1 | 4 | 2 | 1 | 2 | 1 | 0 | 4 | 2 | 1 | 4 | 4 | 1 | 1 | 2 |

**S3 Table. Raw data used for Fig 1C.**

| **Group** | **Chondrocyte number per cartilage area (#/mm^2^)** | | | | | | | | | | | | | | |
| --- | --- | --- | --- | --- | --- | --- | --- | --- | --- | --- | --- | --- | --- | --- | --- |
| **Control** | 1262 | 1209 | 1428 | 1536 | 1321 | 1315 | 1302 | 1812 | 1237 | 1830 | 1478 |  |  |  |  |
| **Palmitate** | 1059 | 1259 | 1242 | 1180 | 1416 | 1326 | 1401 | 1295 | 1417 | 1274 | 468 | 1242 |  |  |  |
| **Oleate** | 992 | 1219 | 1739 | 1362 | 1064 | 1595 | 2036 | 1353 | 1867 | 1717 | 1517 | 1529 | 2003 | 1931 | 914 |

**S4 Table. Raw data used for Fig 1D.**

| **Group** | **Synovitis score** | | | | | | | | | | | | | | |
| --- | --- | --- | --- | --- | --- | --- | --- | --- | --- | --- | --- | --- | --- | --- | --- |
| **Control** | 0 | 0 | 0 | 0 | 0 | 0 | 0 | 1 | 0 | 0 | 0 | 0 | 0 | 0 | 0 |
| **Palmitate** | 1 | 0 | 0 | 1 | 0 | 1 | 1 | 1 | 1 | 1 | 1 | 0 | 0 | 1 |  |
| **Oleate** | 0 | 0 | 1 | 0 | 1 | 0 | 1 | 0 | 0 | 1 | 0 | 0 | 0 | 0 | 0 |

**S5 Table. Raw data used for Fig 5A.**

| **Group** | | **Relative units (pg/ml) for IL-6** | | | | | | | | |
| --- | --- | --- | --- | --- | --- | --- | --- | --- | --- | --- |
| **Pre-Control** | | 9237.73 | 5277.47 | 5769.24 | 4849.76 | 4966.23 | 4663.23 | 7059.47 | 10243.96 | 11824.72 |
| **Post-** | **Control** | 11075.22 | 5616.48 | 6256.49 |  |  |  |  |  |  |
|  | **Palmitate** |  |  |  | 14392.47 | 8461.72 | 12000.98 |  |  |  |
|  | **Oleate** |  |  |  |  |  |  | 10225.47 | 16304.47 | 10205.21 |

**S6 Table. Raw data used for Fig 5B.**

| **Group** | | **Relative units (pg/ml) for IL-10** | | | | | | | | |
| --- | --- | --- | --- | --- | --- | --- | --- | --- | --- | --- |
| **Pre-Control** | | 1779.59 | 1837.92 | 1850.87 | 1672.67 | 1751.65 | 1704.64 | 1883.14 | 1965.59 | 1771.41 |
| **Post-** | **Control** | 2746.78 | 1893.12 | 1737.11 |  |  |  |  |  |  |
|  | **Palmitate** |  |  |  | 3046.01 | 3738 | 2983.04 |  |  |  |
|  | **Oleate** |  |  |  |  |  |  | 1707.59 | 3121.04 | 1994.59 |

**S7 Table. Raw data used for Fig 5C.**

| **Group** | | **Relative units (pg/ml) for TNF-α** | | | | | | | | |
| --- | --- | --- | --- | --- | --- | --- | --- | --- | --- | --- |
| **Pre-Control** | | 3382.27 | 3202.02 | 3358.03 | 2788.58 | 2930.26 | 2827.02 | 3147.28 | 3232.79 | 2875.54 |
| **Post-** | **Control** | 3935.77 | 3230.75 | 2876.51 |  |  |  |  |  |  |
|  | **Palmitate** |  |  |  | 3726.27 | 3740.26 | 3345.53 |  |  |  |
|  | **Oleate** |  |  |  |  |  |  | 2848.52 | 4242.56 | 3431.27 |

**S8 Table. Raw data used for Fig 5D.**

| **Group** | | **Relative units (pg/ml) for IFN-γ** | | | | | | | | |
| --- | --- | --- | --- | --- | --- | --- | --- | --- | --- | --- |
| **Pre-Control** | | 3100.81 | 2687.05 | 2660.84 | 2860.27 | 2637.31 | 3066.75 | 3114.54 | 2649.3 | 2626.28 |
| **Post-** | **Control** | 3606.26 | 2128.58 | 2747.29 |  |  |  |  |  |  |
|  | **Palmitate** |  |  |  | 3471.53 | 2583.57 | 3339.78 |  |  |  |
|  | **Oleate** |  |  |  |  |  |  | 3569.06 | 6742.02 | 2375.63 |

**S9 Table. Raw data used for Fig 5E.**

| **Group** | | **Relative units (pg/ml) for IL-1β** | | | | | | | | |
| --- | --- | --- | --- | --- | --- | --- | --- | --- | --- | --- |
| **Pre-Control** | | 8249.49 | 8068.47 | 7483.98 | 6808.75 | 6625.24 | 7181.23 | 7068.76 | 7945.74 | 7071.99 |
| **Post-** | **Control** | 9435.48 | 4833.25 | 3823.51 |  |  |  |  |  |  |
|  | **Palmitate** |  |  |  | 10448.22 | 3246.01 | 6491.99 |  |  |  |
|  | **Oleate** |  |  |  |  |  |  | 4676.52 | 4608.26 | 6220.74 |

**S10 Table. Raw data used for Fig 5F.**

| **Group** | | **Relative units (pg/ml) for IL-12p70** | | | | | | | | |
| --- | --- | --- | --- | --- | --- | --- | --- | --- | --- | --- |
| **Pre-Control** | | 13019.72 | 13132.98 | 11295.22 | 11420.72 | 11015.72 | 12729.96 | 13439.71 | 12446.72 | 11420.72 |
| **Post-** | **Control** | 12524.21 | 11309.22 | 10534.72 |  |  |  |  |  |  |
|  | **Palmitate** |  |  |  | 11328.48 | 11895.72 | 11038.97 |  |  |  |
|  | **Oleate** |  |  |  |  |  |  | 12121.72 | 9896.23 | 9792.47 |

**S11 Table. Raw data used for Fig 5G.**

| **Group** | | **Relative units (pg/ml) for IL-17** | | | | | | | | |
| --- | --- | --- | --- | --- | --- | --- | --- | --- | --- | --- |
| **Pre-Control** | | 2526.82 | 2517.07 | 2718.56 | 2542.54 | 2562.08 | 2337.05 | 2436.57 | 2292.06 | 2443.76 |
| **Post-** | **Control** | 2072.07 | 2370.79 | 2468.08 |  |  |  |  |  |  |
|  | **Palmitate** |  |  |  | 2954.57 | 2142.09 | 2457.04 |  |  |  |
|  | **Oleate** |  |  |  |  |  |  | 2165.59 | 2249.33 | 2361.85 |
